# Poisoning by Camphor

**Published:** 1875-10

**Authors:** 


Poisoning by Camphor.—Nine well-authenticated cases of poisoning by the homœopathic solution of camphor have been published from time to time in the *British Medical Journal.* This solution contains about a grain of camphor to every two drops, and in doses of fifteen drops and upwards it acts as a strong poison. Some doubt having been expressed by a physician whether camphor was really the cause of the symptoms in the cases reported, from the fact that he had taken the drug in three-grain doses, with no other than a beneficial effect, the *British Medical Journal* replies: "It is a well-known fact that the action of a poison is, *cœteris paribus*, in direct proportion to its solubility. Camphor being very insoluble in the fluids of the canal when given in the form of a dry solid, is in great part eliminated without being absorbed, and has little medicinal or poisonous effect; but when taken in the form of a spirituous solution it is much more readily absorbed, and is, in a corresponding degree, more active."—*New Remedies.*

